# Classification Algorithm for Person Identification and Gesture Recognition Based on Hand Gestures with Small Training Sets

**DOI:** 10.3390/s20247279

**Published:** 2020-12-18

**Authors:** Krzysztof Rzecki

**Affiliations:** AGH University of Science and Technology, 30 Mickiewicz Ave., 30-059 Kraków, Poland; krz@agh.edu.pl

**Keywords:** biometrics, classification, gesture recognition, one-shot learning, person identification, small training sets

## Abstract

Classification algorithms require training data initially labelled by classes to build a model and then to be able to classify the new data. The amount and diversity of training data affect the classification quality and usually the larger the training set, the better the accuracy of classification. In many applications only small amounts of training data are available. This article presents a new time series classification algorithm for problems with small training sets. The algorithm was tested on hand gesture recordings in tasks of person identification and gesture recognition. The algorithm provides significantly better classification accuracy than other machine learning algorithms. For 22 different hand gestures performed by 10 people and the training set size equal to 5 gesture execution records per class, the error rate for the newly proposed algorithm is from 37% to 75% lower than for the other compared algorithms. When the training set consists of only one sample per class the new algorithm reaches from 45% to 95% lower error rate. Conducted experiments indicate that the algorithm outperforms state-of-the-art methods in terms of classification accuracy in the problem of person identification and gesture recognition.

## 1. Introduction

Classification algorithms, an important tool in Computational Intelligence Methods and Statistical learning [1,2,3], are widely used in many areas, for example biometrics, economic trend analysis, human-computer interfaces, medical diagnostics, etc. These methods include random forest [3], *k*-nearest neighbour (*k*NN) [4], probabilistic neural network (PNN) [5], multi-layer perceptron (MLP) [6], support vector machine (SVM) [7], Gaussian processes [8], adaptive neuro-fuzzy inference system (ANFIS) [9], decision trees [3], radial basis function-based neural network (RBF NN) [10], generalized regression neural network (GRNN) [5] as well as siamese neural network (SNN) [11].

The common way to construct a person identification or gesture recognition system based on hand gestures is to collect, with the respect to overfitting, as a large database, as required to reach satisfactory values of the classification coefficients accompanying the receiver operating characteristic (ROC) curve. During the research, the system development or the deploying in real application, when users are volunteers, collecting gestures for predefined gesture recognition tasks is limited only by technical or algorithmic capabilities. In the case of person identification or personalized gesture recognition systems for use in real life it is not so obvious. First of all, the real user may not be so patient, or may be an elder person, or be a disabled person which limits the possibility to record a large number of repetitions of a single gesture, and then the biometric acceptance factor [12] might be lowered. Secondly, in the latest literature [13,14,15,16,17] the researchers point out the necessity to develop customizable gesture recognition systems, where a user can define her/his own gestures. In those systems the small training sets will allow for much quicker introduction of new gestures to be recognized and offer a potential for better user experience in end-user gesture customization. Gesture customization is also important for the most of motor impaired people having heavy movement constraints who for this reason may not be able to perform certain gestures defined by the manufacturer of the system [18,19]. In those systems the small training sets prepared individually for each user will allow the system to be used at all. The small training sets in gesture recognition research approach was already investigated, e.g., for spiking neural networks algorithm [20] and hand gesture recognition with a depth sensor concept [21].

The main contribution of the paper is a novel time series classification algorithm for person identification and gesture recognition where classification model is built using training sets containing a very limited number of gesture repetitions. The algorithm is based on *k*-means and *k*NN algorithms and comparisons based on the vector space model (VSM) [22].

The algorithm was tested and compared to other ones in the exemplary area of human-computer interaction based on hand gestures. A typical human-computer communication using hand glove gestures [23] can be split into two stages: person identification or verification to get access to a computer system and then the gesture recognition to issue commands for this system. Data acquisition for these tasks can be performed using a specialized hand glove [23], which records gestures as time series of data from sensors mounted on it, like accelerometers, gyroscopes and fingers flexion measurement.

The raw data from gesture recordings devices may not be directly suitable for classification or have noisy features causing low classification accuracy. There are some works where authors develop preprocessing methods to improve classification algorithms, for example by using specialized feature extraction algorithms [24] or by applying functional statistical methods [25,26,27]. The emotional state of the person performing a gesture is another source of variability that should be accounted for [28]. There is also some work on using other modalities for gesture recognition, for example vision-based systems [29,30], touchscreen-based methods [31], impedance tomography [32,33], micro-Doppler signatures [34] or controllers like Kinect [35] or LeapMotion [36,37].

Section 2 describes the mathematical model of the data used to present in Section 3 the new algorithm. Section 4 shows the design of the experiments conducted for this study. The results are in Section 5 and they are discussed in Section 6. Finally, Section 7 contains conclusions.

## 2. Mathematical Model

A data sample can be described as a multivariate time series of a number of variables. Values of the variables at the same moment in time constitute an observations. I assume that a data set of such samples is given. The samples are attributed to classes distinguishable by unknown characteristics of the samples. The following description of the algorithm assumes for clarity that samples have an equal number of observation but it can be easily extended to samples having a varying number of observations.

The mathematical model for data representation is described by the following symbols:

- *V*—number of variables
- *v*—variable index (1,2,…,V)
- *D*—number of observations in a sample
- *i*—observation index in a sample (1,2,…,D)
- *J*—number of all samples in the data set
- *j*—sample index in data set (1,2,…,J)
- $x_{j,i}^{v}$—the value of the *i*th observation of *v*th variable and *j*th sample.
- *C*—number of classes
- c(j)—label of the class the *j*th sample belongs to (1,2,…,C)

### 2.1. Data Definition

An observation is a list of values of all variables at the same moment in time and is defined as:

(1) $$\Theta_{j,i}={\left(x_{j,i}^{v}\right)}_{v=1}^{V}=\left(x_{j,i}^{1},x_{j,i}^{2},\ldots ,x_{j,i}^{V}\right).$$

A single sample consists of multiple observations as in Equation (2) read at regular time intervals and is represented by a matrix:

(2) $$\Pi_{j}=\left[\begin{array}{cccc} x_{j,1}^{1} & x_{j,1}^{2} & \ldots & x_{j,1}^{V}\\ x_{j,2}^{1} & x_{j,2}^{2} & \ldots & x_{j,2}^{V}\\ \vdots & \vdots & \ddots & \vdots \\ x_{j,D}^{1} & x_{j,D}^{2} & \ldots & x_{j,D}^{V} \end{array}\right].$$

Each row of the matrix $\Pi_{j}$ corresponds to observation while each column represents a different variable. This notation is used to describe the algorithm presented in this work.

### 2.2. Data Sets

Using the data sets of samples described in Section 2.1 we can define a classification problem which consists of assigning a new sample to one of the predefined classes.

The data set for this task is given by:

(3) $$T=\{\left(\Pi_{j},c\left(j\right)\right):j\in \left\{1,2,\ldots ,J\right\}\}$$

The set of indices of the training samples which are used to build classification function is denoted by TR in the description of the new algorithm.

Then, the classification of an unknown sample is performed by the learned classification function:

(4) f:F→{1,2,…,C},

where *F* is the feature space, that is the set of real matrices with *D* rows and *V* columns.

## 3. New Algorithm

The description of the new classification algorithm is divided into training and predicting phases. We assume that all samples contain an equal number of observations with equal time intervals between observations, thus the continuous time domain is discretized at a fixed number of regularly spaced points of time.

Due to their size, pseudocodes describing both phases of the new algorithm have been included as Supplementary Materials.

### 3.1. Training

Training is a step consisting of building a classification model using training data set TR.

*Step 1. Calculation of signal value differences*

Observations described by Equation (1) are extended by *V* new variables. Values of new variables are calculated as difference of actual (at time index *i*) and previous (at time index i−1) variable values, except the first observation of each sample which is extended by zeros. As a result, we get observations containing 2V variables:

(5) $$\begin{array}{c} \Theta_{j,1}^{\prime }=\left(x_{j,1}^{1},x_{j,1}^{2},\ldots x_{j,1}^{V},\underset{V\;\mathrm{times}}{\underbrace{0,0,\ldots ,0}}\right) \end{array}$$

and

(6) $$\begin{array}{c} \begin{array}{cc} \Theta_{j,i}^{\prime }= & (x_{j,i}^{1},x_{j,i}^{2},\ldots ,x_{j,i}^{V},\\ \; & x_{j,i}^{1}-x_{j,i-1}^{1},x_{j,i}^{2}-x_{j,i-1}^{2},\ldots ,x_{j,i}^{V}-x_{j,i-1}^{V}) \end{array} \end{array}$$

for i∈{2,3,…,D} and j∈TR. Finally we get a matrix:

(7) $$\begin{array}{cc} \Pi_{j}^{\prime }=\left[\begin{array}{cccc} x_{j,1}^{1} & x_{j,1}^{2} & \ldots & x_{j,1}^{S}\\ x_{j,2}^{1} & x_{j,2}^{2} & \ldots & x_{j,2}^{S}\\ \vdots & \vdots & \ddots & \vdots \\ x_{j,D}^{1} & x_{j,D}^{2} & \ldots & x_{j,D}^{S} \end{array}\right. & \\ & \left.\begin{array}{cccc} 0 & 0 & \ldots & 0\\ x_{j,2}^{1}-x_{j,1}^{1} & x_{j,2}^{2}-x_{j,1}^{2} & \ldots & x_{j,2}^{S}-x_{j,1}^{S}\\ x_{j,3}^{1}-x_{j,2}^{1} & x_{j,3}^{2}-x_{j,2}^{2} & \ldots & x_{j,3}^{S}-x_{j,2}^{S}\\ \vdots & \vdots & \ddots & \vdots \\ x_{j,D}^{1}-x_{j,D-1}^{1} & x_{j,D}^{2}-x_{j,D-1}^{2} & \ldots & x_{j,D}^{S}-x_{j,D-1}^{S} \end{array}\right]. \end{array}$$

*Step 2. Merging of data for clustering*

Training samples are merged one-by-one into one long single matrix. This matrix has 2V columns. The number of rows is equal to the product of the size of the training set and the number of observations in a sample.

*Step 3. k-means clustering*

The vector quantization using *k*-means clustering with the given parameter *k* (denoted $k_{1}$ hereafter) is performed over sequences $\Theta_{j,i}^{\prime }$ for observation index i∈{1,2,…,D} and sample number j∈TR. This clustering partitions observations collected in the previous step into *k* clusters. As a result we have a sequence Ω of symbols representing partitions (clusters),

(8) $$\Omega ={\left(\omega_{l}\right)}_{l=1}^{k_{1}},$$

where $\omega_{l}$ is the symbol representing the *l*th cluster. Each symbol $\omega_{l}$ for $l\in \{1,2,\ldots ,k_{1}\}$ has a corresponding set of coordinates $\Theta_{\omega_{l}}^{\prime }$. The coordinates are from the same space as input data, the $\Theta_{i,j}^{\prime }$ sequences defined by Equations (5) and (6).

Symbols from Equation (8) are assigned to observations $\Theta_{j,i}^{\prime }$. For each i∈{1,2,…,D} and j∈TR the symbol $\omega_{\Theta_{j,i}^{\prime }}$ represents the cluster the observation $\Theta_{j,i}^{\prime }$ belongs to. Each observation $\Theta_{j,i}^{\prime }$ is assigned to the nearest cluster calculated using a certain distance function $d_{clust}$.

The set of the features $\Theta^{\prime }$ and the corresponding classes Ω are a training input to *k*NN classifier used in prediction phase Step 2.

*Step 4. Replacing observations by symbols*

Each observation $\Theta_{j,i}^{\prime }$ in each training sample (indicated by *j* and *i*) is replaced by a symbol corresponding to the cluster the observation belongs to, $\omega_{\Theta_{j,i}^{\prime }}$. As a results, training samples are represented by sequences of symbols:

(9) $$\Pi_{j}^{\prime \prime }={\left(\omega_{\Theta_{j,i}^{\prime }}\right)}_{i=1}^{D}$$

for j∈TR.

*Step 5. Calculating the frequency table*

For each training sample indexed by j∈TR the frequency sequence, known as the Vector Space Model [22], is computed using the corresponding sequence of symbols $\Pi_{j}^{\prime \prime }$:

(10) $$B_{j}={\left(\beta_{j,l}\right)}_{l=1}^{k_{1}}$$

where $\beta_{j,l}$ is the number of times the symbol $\omega_{l}$ appears in the sequence $\Pi_{j}^{\prime \prime }$.

*Step 6. Calculating the class centroid*

Within each of class c∈{1,2,…,C}, the mean frequency value of each symbol is calculated, as

(11) $$\Gamma_{c}={\left(\frac{1}{\#S_{c}}\sum_{j\in S_{c}}\beta_{j,l}\right)}_{l=1}^{k_{1}},$$

where $S_{c}$ is the set of indices of samples from the training set assigned to the class *c* and $\#S_{c}$ is the number of elements in set $S_{c}$. These class centroids represent the model of the classification algorithm.

### 3.2. Prediction

A new sample represented by a matrix $\Pi_{new}$ is assigned to one of the classes using the model built by the described algorithm. Following steps need to be performed during classification:

*Step 1. Difference of signal values*

Each observation of the new sample is extended as described in step 1 of the training procedure. The resulting matrix is denoted $\Pi_{new}^{\prime }$ and is computed analogically to $\Pi_{j}^{\prime }$ in Equation (7).

*Step 2. Replacing observations by symbols*

Extended observations of a new sample collected in the matrix $\Pi_{new}^{\prime }$ are replaced by symbols from the sequence Ω defined by Equation (8) developed during the training phase. This step is performed using the *k*NN (*k*-Nearest Neighbor) algorithm. The value of the *k* parameter is denoted $k_{2}$ and the distance function is denoted $d_{kNN}$ hereafter. The training data for this algorithm consist of observations $\Theta_{j,i}^{\prime }$ for all j∈TR and i∈{1,2,…,D} with corresponding symbols $\omega_{\Theta_{j,i}^{\prime }}$ used as classes for the purpose of training the *k*NN model.

The matrix $\Pi_{new}^{\prime }$ is transformed to a sequence of symbols $\Pi_{new}^{\prime \prime }$ corresponding to sequences calculated in step 4 of the training phase. They are, however, obtained by classifying each row of $\Pi_{new}^{\prime }$ using the learned *k*NN model.

*Step 3. Calculate the frequency table*

Calculate the symbol frequency table in VSM (Vector Space Model) of the new sample as in step 5 of the training phase:

(12) $$B_{new}={\left(\beta_{new,l}\right)}_{l=1}^{k_{1}}$$

where $\beta_{new,l}$ is the number of times the symbol $\omega_{l}$ appears in the sequence $\Pi_{new}^{\prime \prime }$.

*Step 4. Indicate the class label of a new survey*

The distances between the symbol frequency table $B_{new}$ of the new sample and centroids $\Gamma_{c}$ of each class c∈{1,2,…,C} from Equation (11) are calculated using a distance function $d_{VSM}$. The index *c* of the nearest centroid indicates the class for the new sample.

### 3.3. Parameter Optimization

The algorithm has a set of parameters that have to be adjusted to optimize its accuracy. For the training phase the number of partitions $k_{1}$ and the distance function $d_{clust}$ of the *k*-means algorithm need to be determined. For the prediction phase, the number of neighbours $k_{2}$ and distance function of the *k*NN algorithm were optimized, as well as the distance function $d_{VSM}$.

Standard distance functions were considered for $d_{clust}$, $d_{kNN}$ and $d_{VSM}$, including the city block distance, Chebyshev distance, correlation distance, cosine distance, Euclidean distance, Hamming distance, Jaccard distance, Mahalanobis distance, Minkowski distance, squared Euclidean distance and the Spearman distance.

Tested and optimal parameters are presented in more detail Section 5.

### 3.4. Time Complexity

The time complexity of the training depends linearly on the product of: the number of variables in an observation, the number of observations in a sample, the number of samples in each class and the number of classes. Scaling up of training depends only on the number of classes, because we consider small (and constant) training sets, as in motivation of this work and rest of the parameters are constant.

The classification time complexity of a single gesture is similar to training, but the number of samples in each of classes is omitted. Scaling up of classification is also linearly dependent only on the number of classes.

## 4. Experiments

The new algorithm presented in this article was tested, evaluated and compared to other methods using a database of gesture execution records [23] available as Supplementary Materials. To build this database the DG5 VHand glove was used. The database was used in two different problems: person identification using a known gesture and gesture recognition assuming the person performing it is identified. Two experiments with evaluation based on data set resampling were performed to compare the new method to well-known algorithms listed in Section 1. The different methods were compared quantitatively by determining the correlation between the training set size and the error rate of classification.

### 4.1. Gestures Data Set

**Figure 1:**
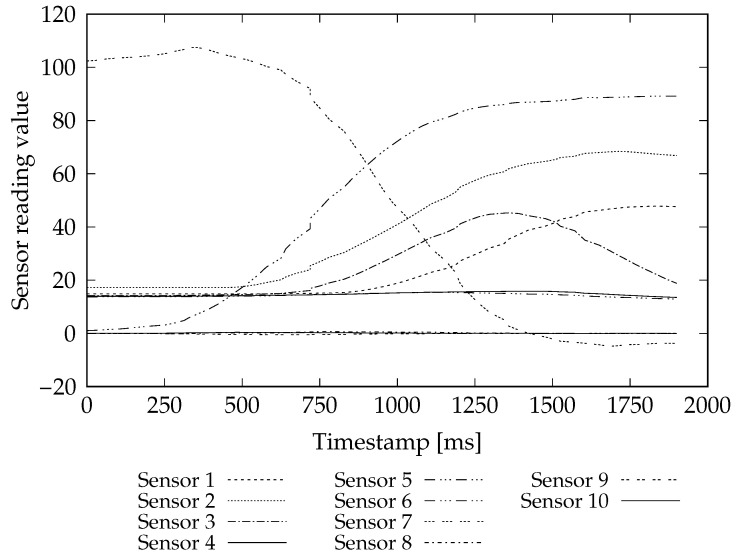
Visualization of exemplary gesture execution signals values.

The glove used to acquire the data has 10 sensors: five finger flexion sensors, one for each of the fingers (thumb, index, middle, ring, little), three accelerometers to measure hand movements in each of *x*-, *y*-, and *z*-axis and two gyroscopes to determine hand orientation (roll and pitch). The sensors are numbered from 1 to 10 in the given order. A single database record, called survey, corresponds to one gesture execution performed by a single person. Surveys are represented by matrices structured as in the example in Table 1. Their rows correspond to sensor readings at a particular moment. The first column denotes timestamp while the other ones correspond to readings from ten glove sensors pulled at that time. Glove readings of a sample survey are visualized in Figure 1.

Each sensor corresponds to a variable, a single reading of all sensors at the same time is an observation and the time series of observations from a particular gesture execution is a sample.

The database consists of surveys of 22 different hand gestures executed 10 times by 10 people, J=2200 records in total. The details of this database are discussed in [23]. A single survey (single gesture execution) contains from 12 to 149 observations and lasts from 360 ms to 4625 ms. Readings are recorded at a sampling rate varying between about 20 Hz to 40 Hz.

### 4.2. Experiment Design

In both experiments, the comparisons of the algorithms were performed separately for each number n∈{1,2,...,S−1} of surveys taken from each class to the training set. For a given *n*, *S* separate samplings from the data set were performed. The samples for the training set ${\mathrm{TR}}_{n,w}$ were selected using a circular sliding window scheme within each class:

(13) $$\begin{array}{c} \begin{array}{cc} {\mathrm{TR}}_{n,w}= & \{j_{c,r}:c\in \{1,2,\ldots ,C\},\\ \; & r\in \{w,w+1,\ldots ,w+n-1\}\}, \end{array} \end{array}$$

where w∈{1,2,…,S} is the resampling number and $j_{c,r}$ is the sample index of the *r*th sample in class *c*, r∈{1,2,…,S}. In Equation (13) it is also assumed that $j_{c,r+S}=j_{c,r}$ for each class c∈{1,2,…,C} and sample number r∈{1,2,…,S}.

The test set consisted of the other S−n samples from each class. The dependency of the error rate on the size *n* of the training set was measured.

### 4.3. Data Preprocessing

Surveys acquired directly from glove are of different length because of differences among gestures shapes and irregular speed of their execution. Additionally, the readings from the glove hardware are not performed at regular time intervals. The preprocessing step is performed to resample the surveys to ensure that each survey is represented by a matrix $\Pi_{j}$ of the same size given by Equation (2) and that time intervals between consecutive readings are constant. This step is performed using linear interpolation method. As a result, every survey record contains exactly the same number of observations, and thus observations can be consistently numbered by an index i∈(1,2,…,D).

For other compared methods it is required additionally to transform the matrices $\Pi_{j}$ to a single-column vector. This is done by concatenating columns of each matrix $\Pi_{j}$ as in the previous work [23].

### 4.4. Methods Evaluation

The new algorithm was compared to the well-known ones listed in Section 1. Each method has various parameters to be adjusted to configure the given algorithm for the best classification results. The optimal parameters were looked for to minimize the mean error rate as algorithm evaluation criteria. The values of these parameters were determined using grids of parameter values and the exhaustive search. The Winner Takes All (WTA) rule was used to indicate the correct class in this multi-class classification problem and all other classes were indicated as incorrect.

Implementation of the test environment was based on Matlab software, scikit-learn Python Library [38] based on SciPy, NumPy and NeuPy, and LIBSVM.

## 5. Results

The model presented in Section 3 was tested using the database described in Section 4.1 in two experiments introduced in Section 4. Both experiments are the classification problems where the single class is a set of samples described in Section 2.1. In the first experiment the new algorithm was compared to other in task of the person identification using one given gesture. There were 22 sub-experiments for each gesture separately. Each of the sub-experiments had 10 classes (C=10) corresponding to 10 people who performed a given gesture. In the second experiment the task of gesture recognition using a gesture performed by the identified person was considered. There were 10 sub-experiments for each person separately. In each-sub experiment there were 22 classes (C=22), one for each gesture type. In both experiments the number of samples per class *S* is equal to 10, and the number of surveys in each of sub experiments is equal to, respectively, 100 and 220.

Proposed algorithm may be a part of a complete system that includes hardware, data acquisition and recording module, classification algorithm, decision module, etc. If a person is doing nothing, the part of the system that processes the signal from hardware should tag this signal as empty and the system should not pass it to the classification algorithm.

### 5.1. Parameter Selection

The PNN algorithm depends on one parameter: the spread. The method was tested with spread in a range from 0.01 to 1.00. No particular value in this range resulted in the highest accuracy in all cases.

The *k*NN method depends on the number of neighbours and the distance function. The number of neighbours was tested in the range from 1 to 4, with 1 neighbour resulting in the most accurate classification. The city block distance was found to be the best on average in terms of classification accuracy but for some particular gestures or persons different distance functions were better.

For the SVM based classification, the core parameters are SVM type and kernel (with its parameters). In the experiments there were tested SVM types: C-SVC, ν-SVC, one-class SVM, ϵ-SVR and ν-SVR and SVM kernel types: linear $k_{lin}\left(x,y\right)=x^{T}y$, polynomial $k_{poly}\left(x,y\right)={\left(x^{T}y+\gamma \right)}^{d}$, Radial Basis Function-based $k_{RBF}=\exp\left(-\gamma {∥x-y∥}^{2}\right)$ and sigmoid $k_{sigm}\left(x,y\right)=\tanh\left(\gamma x^{T}y+C\right)$. The C-SVC variant with the polynomial kernel was the most accurate among variants of the SVM classifier. The degree *d* of the polynomial kernel was tested in the range from 1 to 5 and γ parameter tested in the range from 0.5 to 1.0.

In the standard multi-layer perceptron neural networks the optimized parameters are the number of neurons and the network optimization algorithm used for training. The number of neurons in the hidden layer was tested in the range from 10 to 100 with the step equal to 10, but the most accurate classification was obtained mostly in the range from 10 to 40 neurons. The hyperbolic tangent activation function was used in the hidden layer and the softmax activation function was used in the output layer. Different optimization algorithms were tested but scaled conjugate gradient and Fletcher-Powell conjugate gradient produced neural networks with the highest accuracy.

The number of trees affected the accuracy of the random forest classifier (denoted TBG) to the greatest degree. It was found that in the studied problems the forest should have at least 50 trees.

The siamese neural network was based on a convolutional neural network and was adapted into one-dimensional data. The architecture consisted of two convolutional layers with ReLU activation function and maximum pooling. Using more than two such layers did not give any relevant improvement. Three parameters were optimized in both convolutional layers: the size of filters in the range from 1 to 10, the number of filters in the range from $2^{5}$ to $2^{8}$, and the filter stride from 1 to the filter size. The best results were achieved when the first layer consisted of 64 filters of size 6 and stride 1 and in the second layer there were 128 filters of size 7 and stride 1 as well. There was also the fully connected layer optimized with the size of features vector in the range from $2^{7}$ to $2^{12}$, with $2^{11}$ resulting in the most accurate classification. As the SNN algorithm needs at least two training samples for each class to learn when samples are similar, the results start also from the number of training samples equal to two.

Finally, the new algorithm (denoted QUA, as it is based mainly on vector *qua*ntization) was tested for parameters listed in Section 3.3 and the most accurate classification was reached for the number of clusters $k_{1}$ in the range from 140 to 200 (tested with step 20), the city block distance function $d_{clust}$, the number of neighbours in the *k*NN algorithm $k_{2}$ equal to 1, the *k*NN distance function $d_{kNN}$ equal to the standardized Euclidean distance and the city block distance used in VSM as $d_{VSM}$.

### 5.2. Efficiency Results

The mean error rate as algorithm evaluation criteria was used:

(14) ERR=1−ACC

where ACC is the number of correctly classified instances divided by the number of all classified instances.

**Figure 2:**
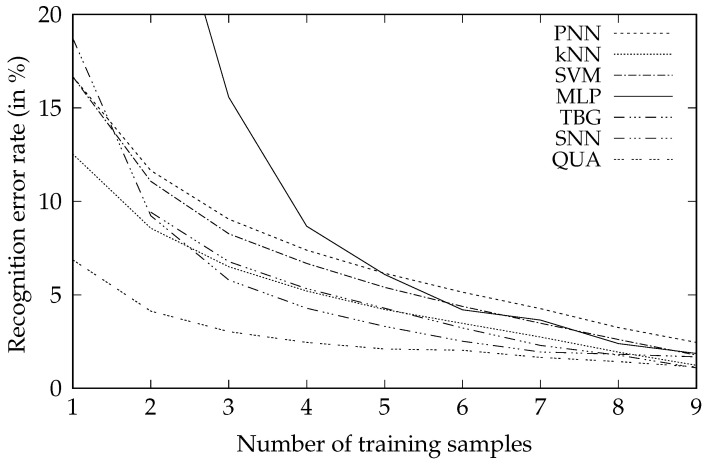
Dependence of the average error rate of different classifiers in the problem of person recognition on the number of training samples per class *n*.

**Figure 3:**
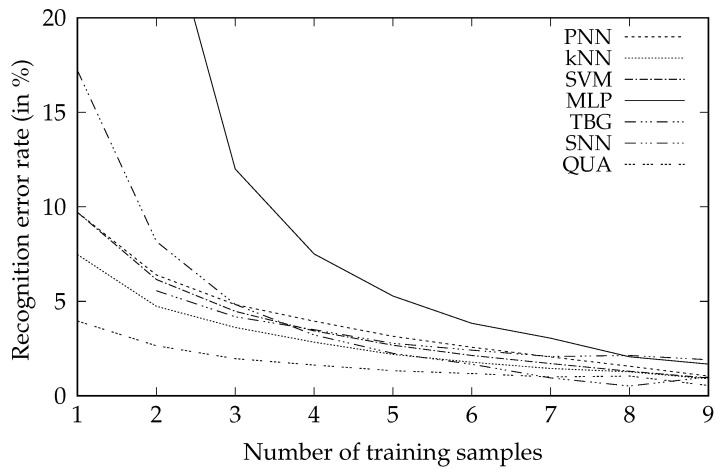
Dependence of the average error rate of different classifiers in the problem of gesture recognition on the number of training samples per class *n*.

The comparison of the new algorithm and other classification methods optimized with respect to the mean error rate there is presented as follows. In Table 2 and correspondingly Figure 2 there are results of the person identification experiment, while in Table 3 and correspondingly Figure 3 there are results of the gesture recognition experiment. Both tables and figures present dependence of the average error rate of different classifiers on the number of training samples per class.

The two confusion matrices were calculated to evaluate the behavior of the proposed algorithm for the most interesting case of using only one training sample from each class. The matrices are attached as Supplementary Materials and the results are as follows. In the case of person identification the most distinguishable gestures executions were performed by person number 3, and the least by persons number 4 and 8. The lowest number of misclassified executions of gestures were assigned to person number 10, where the most misclassifications were assigned to person number 2. In the case of gesture recognition the most distinguishable gestures have numbers 11 and 19, and the least distinguishable were 7 and 21. Misclassfied gestures were most commonly recognized as gesture 15 and least often as gesture number 11.

### 5.3. Performance Results

Training and testing times of the new algorithm and other existing algorithms were compared. To benchmark algorithms a computer running Linux Mint 20 Ulyana with Intel(R) Core(TM) i7-9700KF CPU @ 3.60 GHz CPU and 32 GB of Crucial DDR4 RAM at 2667 MT/s was used. The SNN implementation was tested using Gigabyte graphic card with GeForce RTX 2070 SUPER graphic processor unit with driver version 455.32.00 and CUDA version 11.1. The results are presented as follows. In Table 4 there is an efficiency comparison of algorithms in the person identification experiment, while in Table 5 there is a comparison in the gesture recognition experiment.

## 6. Discussion

Both experiments that were performed for the tasks of person identification and gesture recognition indicated that the new classifier provides higher accuracy than all other well-known algorithms. The difference is the most significant when the number of training samples is small, up to about 6 to 9 samples per class.

In addition to methods listed in Section 1 some other ones were also tested. For methods like Gaussian process classifier, an adaptive neuro-fuzzy inference system, decision trees, radial basis function-based neural networks and generalized regression neural networks either the computation time or achieved results were not sufficient to reach fully comparable results and thus they are not discussed in more detail.

It can be observed that the new algorithm results in the most accurate classification when the 20-dimensional extended observation space is quantized into a relatively large number of clusters. Depending on the problem, there are either 2100 or 4620 points grouped into from 140 to 200 clusters. This step reduces a continuous-variable classification problem into a discrete one which is one of the sources of generalization power of the proposed method. It is also notable that the algorithm has only five discrete free parameters ($k_{1}$, $k_{2}$, $d_{clust}$, $d_{kNN}$ and $d_{VSM}$), which makes overfitting less likely then in most classification methods.

The data is further reduced in steps 5 of 6 of the training phase, where the frequency table approach is used. It reduces the need to perform curve registration by only considering how long the gesture execution stayed in a particular discrete state. The temporal component of the data is however still present in the form of the difference components calculated in step 1 of training and prediction procedures.

The experiments have shown that the time taken by the training phase of the new algorithm was average compared to benchmark results of other algorithms. The classification time was not the longest among all algorithms however, but substantially longer than the fastest one. The main bottleneck of the new classification algorithm is located in Step 2. where replacing observations are replaced by symbol. For this step the *k*NN algorithm is used. The shortest recorded survey (gesture execution) lasts 360 ms and the mean classification time of about 11 ms to 13 ms is less than 4% of it. In real applications of person identification or gesture recognition this classification time should not be noticeable. However, future work on the new algorithm should be concentrated on improving the performance of Step 2.

## 7. Conclusions

In this article the new time series classification algorithm was presented. The algorithm is based on vector quantization of recorded observations, transforming them into sequences of discrete symbols comparing them using a vector space model. The algorithm was tested, evaluated and compared to state-of-the-art methods using hand gesture recordings in tasks of person identification and gesture recognition. It was shown that the new algorithm is more accurate than other methods, especially on small training sets.

## Figures and Tables

**Table 1:** Gesture execution example (survey).

		$\overset{\;\;\;\;\;\;\;}{\leftarrow }$ Variables $\overset{\;\;\;\;\;\;\;}{\to }$				
	Timestamp	Sensor 1	Sensor 2	Sensor 3	…	Sensor 10
Observations	0	14.8438	17.1875	14.2725	…	0.0343
	47	14.8438	17.1875	14.2529	…	0.0467
	63	14.8438	17.1875	14.2432	…	0.0513
	…	…	…	…	…	…
	1869	47.7539	67.0801	20.3076	…	−0.0044
	1900	47.6465	66.8164	18.8184	…	0.0010

**Table 2:** Person recognition error rate. The first column, labelled *n*, denotes the number of training samples per class. The best result in a given row is shown in bold and indicates the least erroneous classifier.

*n*	PNN	kNN	SVM	MLP	TBG	SNN	QUA
1	16.67%	12.53%	16.67%	77.24%	18.72%	—	**6.87%**
2	11.65%	8.56%	11.07%	29.95%	9.23%	9.42%	**4.13%**
3	9.05%	6.50%	8.27%	15.57%	5.79%	6.78%	**3.03%**
4	7.39%	5.20%	6.68%	8.67%	4.28%	5.33%	**2.45%**
5	6.15%	4.21%	5.40%	6.09%	3.31%	4.28%	**2.10%**
6	5.14%	3.47%	4.38%	4.20%	2.52%	3.23%	**2.03%**
7	4.26%	2.74%	3.48%	3.65%	1.94%	2.29%	**1.65%**
8	3.25%	1.93%	2.61%	2.39%	1.82%	1.77%	**1.43%**
9	2.45%	1.23%	1.77%	1.86%	1.68%	1.09%	**1.14%**

**Table 3:** Gesture recognition error rate. The first column, labelled *n*, denotes the number of training samples per class. The best result in a given row is shown in bold and indicates the least erroneous classifier.

*n*	PNN	kNN	SVM	MLP	TBG	SNN	QUA
1	9.71%	7.47%	9.71%	79.23%	17.21%	—	**3.96%**
2	6.40%	4.74%	6.16%	27.26%	8.17%	5.56 %	**2.65%**
3	4.83%	3.62%	4.47%	12.00%	4.84%	4.18 %	**1.97%**
4	3.95%	2.84%	3.44%	7.51%	3.22%	3.50 %	**1.63%**
5	3.15%	2.19%	2.68%	5.28%	2.25%	2.78 %	**1.33%**
6	2.57%	1.78%	2.14%	3.84%	1.67%	2.42 %	**1.18%**
7	2.06%	1.45%	1.71%	3.05%	**0.95%**	2.08 %	1.00%
8	1.57%	1.27%	1.30%	2.07%	**0.52%**	2.14 %	1.05%
9	1.05%	0.91%	0.95%	1.68%	1.00%	1.91 %	**0.55%**

**Table 4:** Algorithm performance for person identification, time in milliseconds. The training time was measured for classes size of 1. The classification time was measured for one sample.

	PNN	*k*NN	SVM	MLP	TBG	SNN	QUA
training	12.75	3.17	0.42	92.20	57.55	59.16 s	11.87
classification	0.0514	0.4819	<0.1 μs	0.0574	0.4290	151.84	10.3926

**Table 5:** Algorithm performance for gesture recognition, time in milliseconds. The training time was measured for classes size of 1. The classification time was measured for one sample.

	PNN	*k*NN	SVM	MLP	TBG	SNN	QUA
training	15.71	4.00	1.92	95.77	61.76	60.74 s	14.93
classification	0.0376	0.5081	<0.1 μs	0.0289	0.2638	104.32	11.50

## Supplementary Materials

The following are available online at https://www.mdpi.com/1424-8220/20/24/7279/s1: pseudocodes of the proposed algorithm, gesture execution records, confusion matrices.
