# Supplementary material for: Classification Algorithm for Person Identification and Gesture Recognition Based on Hand Gestures with Small Training Sets
Source: Sensors (Basel). 2020 Dec 18;20(24):7279. doi: 10.3390/s20247279 (PMC7766068; doi:10.3390/s20247279)
Supplement: Supplementary file 1 [file sensors-20-07279-s001.zip › sensors-988263-for XML-supp/article-supplement-algorithm-pseudocodes.pdf]

Supplementary material for the paper Classification Algorithm for Person Identification and Gesture Recognition Based on Hand Gestures with Small Training Sets

(Dated: December 3rd, 2020)

Below there are pseudocodes of the algorithm proposed in the article.

Require: a survey $\Theta_i = (x_i^v)_{v=1}^V$, $i \in \{1, 2, \ldots, D\}$

for $v = 1$ **to** $2V$ **do**
  if $v \leq V$ **then**
    $\Theta'(1, v) \leftarrow x_1^v$
  else
    $\Theta'(1, v) \leftarrow 0$
  end if
end for
for $i \in \{2, 3, \ldots, D\}$ **do**
  for $v \in \{1, 2, \ldots, 2V\}$ **do**
    if $v \leq V$ **then**
      $\Theta'(i, v) \leftarrow x_i^v$
    else
      $\Theta'(1, v) \leftarrow x_i^v - x_{i-1}^v$
    end if
  end for
end for
return Θ'

Algorithm 1: Transformation, step 1 in training and prediction phases.

Require: symbol table $\Pi''(i)$, $i \in \{1, 2, \ldots, D\}$

for $l = 1$ **to** k_1 **do**
  $B(l) \leftarrow 0$
end for
for $i = 1$ **to** D **do**
  $B(\Pi''(i)) \leftarrow B(\Pi''(i)) + 1$
end for
return B

Algorithm 2: Frequency table calculation, step 5 in training and step 3 in prediction.

Require: training data $\Theta_{j,i} = (x^v_{j,i})^V_{v=1}$, c_j, $j \in TR$, $i \in \{1, 2, \ldots, D\}$; clustering parameters k_1, d_{clust}; kNN parameters k_2, d_{kNN}

{Step 1.}
for $j \in TR$ **do**
  $\Theta'(J, :, :) \leftarrow$ TRANSFORM$(\Theta_{j,:})$
end for
{Step 2.}
for $j \in TR$ **do**
  for $i = 1$ **to** D **do**
    for $v = 1$ **to** $2V$ **do**
      $\Theta''(D(j-1) + i, v) \leftarrow \Theta'(j, i, v)$
    end for
  end for
end for
{Step 3.}
$\Omega \leftarrow$ K_MEANS$(\Theta'', k_1, d_{clust})$
{Step 4.}
for $j \in TR$ **do**
  for $i = 1$ **to** D **do**
    $\Pi''(i, j) \leftarrow 1$
    $\mathit{mindist} \leftarrow d_{clust}(\Omega(1), \Theta'(j, i, :))$
    for $c \in \{2, 3, \ldots, k_1\}$ **do**
      $\mathit{curdist} \leftarrow d_{clust}(\Omega(c), \Theta'(j, i, :))$
      if $\mathit{curdist} < \mathit{mindist}$ **then**
        $\Pi''(i, j) \leftarrow c$
        $\mathit{mindist} \leftarrow \mathit{curdist}$
      end if
    end for
  end for
end for
{Step 5.}
for $j \in TR$ **do**
  $B(j, :) \leftarrow$ FREQ_TABLE$(\Pi''(:, j))$
end for
{Step 6.}
for $l = 1$ **to** k_1 **do**
  for $c = 1$ **to** C **do**
    $\Gamma(c, l) \leftarrow 0$
    $N \leftarrow 0$
    for $j \in TR$ **do**
      if $c_j = c$ **then**
        $N \leftarrow N + 1$
        $\Gamma(c, l) \leftarrow \Gamma(c, l) + B(j, l)$
      end if
    end for
    $\Gamma(c, l) \leftarrow \Gamma(c, l)/N$
  end for
end for
{kNN training with features Θ', classes Ω, k parameter equal to k_2 and distance function d_{kNN}.}
KNN_MODEL $\leftarrow$ KNN_TRAINING$(\Theta', \Omega, k_2, d_{kNN})$
return Γ, KNN_MODEL

Algorithm 3: Training algorithm.

Require: new survey $\Theta_i = (x_i^v)_{v=1}^V$, $i \in \{1, 2, \ldots, D\}$; centroids $\Gamma(c, l)$, $c \in \{1, 2, \ldots, C\}$, $l \in \{1, 2, \ldots, k_1\}$; clustering parameter k_1; kNN model; distance function d_{VSM}

{Step 1.}
$\Theta' \leftarrow \textsc{transform}(\Theta_i)$
{Step 2.}
for $i = 1$ **to** D **do**
    $\Pi''_{new}(i) \leftarrow \textsc{knn\_predict}(\textsc{knn\_model},\ \Theta'_i)$
end for
{Step 3.}
$B_{new} \leftarrow \textsc{freq\_table}(\Pi''_{new})$
{Step 4.}
$c_{new} \leftarrow 1$
$mindist \leftarrow d_{VSM}(\Gamma(1, :), B_{new})$
for $c \in \{2, 3, \ldots, C\}$ **do**
    $curdist \leftarrow d_{VSM}(\Gamma(c, :), B_{new})$
    if $curdist < mindist$ **then**
        $c_{new} \leftarrow c$
        $mindist \leftarrow curdist$
    end if
end for
return c_{new}

Algorithm 4: Prediction algorithm.
