# Supplementary material for: Classification Algorithm for Person Identification and Gesture Recognition Based on Hand Gestures with Small Training Sets
Source: Sensors (Basel). 2020 Dec 18;20(24):7279. doi: 10.3390/s20247279 (PMC7766068; doi:10.3390/s20247279)
Supplement: Supplementary file 1 [file sensors-20-07279-s001.zip › sensors-988263-for XML-supp/article-supplement-confusion-matrices.docx]

Supplementary material for the paper “Classification Algorithm for Person Identification and Gesture Recognition Based on Hand Gestures with Small Training Sets”

Below there is the confusion matrix related to the person identification experiment. The 10-fold cross validation was performed, the trainings were performed using sequentially the only one sample of each of classes.

| **True class (person)** | | | |  |  |  |  |  |  |  |
| --- | --- | --- | --- | --- | --- | --- | --- | --- | --- | --- |
|  | **Predicted class (person)** | | | | | | | | | |
|  | **1** | **2** | **3** | **4** | **5** | **6** | **7** | **8** | **9** | **10** |
| **1** | 1756 | 54 | 53 | 3 | 28 | 32 | 18 | 33 | 1 | 2 |
| **2** | 8 | 1880 | 21 | 5 | 38 | 5 | 16 | 7 | 0 | 0 |
| **3** | 11 | 23 | 1906 | 6 | 11 | 4 | 12 | 2 | 5 | 0 |
| **4** | 26 | 51 | 42 | 1723 | 23 | 67 | 33 | 4 | 8 | 3 |
| **5** | 5 | 53 | 16 | 14 | 1866 | 8 | 14 | 4 | 0 | 0 |
| **6** | 11 | 7 | 15 | 3 | 33 | 1875 | 11 | 25 | 0 | 0 |
| **7** | 11 | 9 | 14 | 1 | 36 | 18 | 1867 | 24 | 0 | 0 |
| **8** | 35 | 51 | 32 | 9 | 28 | 41 | 64 | 1689 | 17 | 14 |
| **9** | 8 | 16 | 14 | 27 | 20 | 7 | 23 | 13 | 1841 | 11 |
| **10** | 12 | 4 | 4 | 15 | 7 | 8 | 6 | 10 | 37 | 1877 |
|  |  |  |  |  |  |  |  |  |  |  |
| Missclasified: | |  |  |  |  |  |  |  |  |  |
|  | 127 | 268 | 211 | 83 | 224 | 190 | 197 | 122 | 68 | 30 |

|  | the highest values |
| --- | --- |
|  | the lowest values |

Below there is the confusion matrix related to the gesture recognition experiment. The cross validation was performed similarly like in case of person identification.

| **True class (gesture)** | | | | | |  |  |  |  |  |  |  |  |  |  |  |  |  |  |  |  |  |  |
| --- | --- | --- | --- | --- | --- | --- | --- | --- | --- | --- | --- | --- | --- | --- | --- | --- | --- | --- | --- | --- | --- | --- | --- |
|  | **Predicted class (gesture)** | | | | | | | | | | | | | | | | | | | | | | |
|  | **1** | **2** | **3** | **4** | **5** | **6** | **7** | **8** | **9** | **10** | **11** | **12** | **13** | **14** | **15** | **16** | **17** | **18** | **19** | **20** | **21** | **22** |  |
| **1** | 863 | 1 | 5 | 11 | 0 | 1 | 0 | 0 | 12 | 0 | 2 | 0 | 0 | 2 | 1 | 1 | 0 | 0 | 0 | 0 | 1 | 0 |  |
| **2** | 6 | 811 | 9 | 1 | 3 | 0 | 3 | 9 | 6 | 1 | 2 | 12 | 1 | 2 | 1 | 0 | 3 | 0 | 4 | 5 | 1 | 20 |  |
| **3** | 8 | 6 | 802 | 2 | 2 | 2 | 7 | 3 | 10 | 17 | 1 | 4 | 6 | 1 | 0 | 3 | 5 | 0 | 1 | 0 | 14 | 6 |  |
| **4** | 11 | 0 | 1 | 844 | 0 | 0 | 4 | 0 | 4 | 0 | 0 | 2 | 0 | 13 | 5 | 10 | 0 | 1 | 3 | 0 | 0 | 2 |  |
| **5** | 1 | 0 | 4 | 0 | 875 | 3 | 4 | 0 | 2 | 2 | 0 | 2 | 0 | 0 | 0 | 4 | 0 | 0 | 2 | 0 | 1 | 0 |  |
| **6** | 1 | 0 | 0 | 0 | 4 | 873 | 0 | 0 | 9 | 4 | 0 | 1 | 0 | 0 | 0 | 1 | 0 | 0 | 1 | 4 | 2 | 0 |  |
| **7** | 0 | 0 | 8 | 1 | 23 | 5 | **793** | 15 | 2 | 19 | 0 | 2 | 16 | 0 | 1 | 0 | 1 | 0 | 2 | 1 | 8 | 3 |  |
| **8** | 1 | 3 | 3 | 1 | 5 | 0 | 27 | 807 | 9 | 5 | 1 | 5 | 1 | 15 | 0 | 0 | 4 | 0 | 5 | 1 | 1 | 6 |  |
| **9** | 0 | 1 | 3 | 0 | 2 | 18 | 1 | 0 | 829 | 19 | 0 | 5 | 9 | 0 | 0 | 2 | 0 | 0 | 0 | 8 | 2 | 1 |  |
| **10** | 0 | 0 | 2 | 0 | 4 | 1 | 1 | 2 | 8 | 881 | 0 | 0 | 0 | 0 | 0 | 1 | 0 | 0 | 0 | 0 | 0 | 0 |  |
| **11** | 0 | 0 | 1 | 0 | 0 | 1 | 0 | 0 | 4 | 0 | **887** | 0 | 0 | 1 | 0 | 4 | 0 | 0 | 1 | 0 | 0 | 1 |  |
| **12** | 0 | 1 | 5 | 0 | 7 | 7 | 0 | 0 | 16 | 3 | 0 | 815 | 1 | 0 | 29 | 2 | 0 | 0 | 8 | 1 | 5 | 0 |  |
| **13** | 0 | 0 | 3 | 1 | 0 | 2 | 1 | 0 | 7 | 11 | 0 | 7 | 830 | 0 | 3 | 0 | 0 | 0 | 0 | 0 | 34 | 1 |  |
| **14** | 0 | 0 | 1 | 13 | 0 | 0 | 0 | 0 | 2 | 0 | 0 | 0 | 1 | 851 | 23 | 4 | 0 | 1 | 0 | 0 | 2 | 2 |  |
| **15** | 0 | 0 | 0 | 3 | 0 | 0 | 0 | 0 | 0 | 0 | 0 | 3 | 3 | 24 | 846 | 0 | 0 | 21 | 0 | 0 | 0 | 0 |  |
| **16** | 0 | 1 | 5 | 4 | 0 | 0 | 0 | 0 | 2 | 0 | 0 | 4 | 0 | 1 | 0 | 861 | 17 | 0 | 0 | 0 | 0 | 5 |  |
| **17** | 0 | 1 | 5 | 0 | 2 | 0 | 1 | 3 | 1 | 2 | 0 | 0 | 0 | 5 | 2 | 19 | 835 | 1 | 23 | 0 | 0 | 0 |  |
| **18** | 0 | 0 | 3 | 1 | 0 | 1 | 0 | 0 | 3 | 0 | 0 | 4 | 0 | 7 | 73 | 0 | 0 | 807 | 0 | 0 | 1 | 0 |  |
| **19** | 0 | 0 | 0 | 0 | 2 | 4 | 0 | 0 | 0 | 4 | 0 | 3 | 0 | 0 | 0 | 1 | 0 | 0 | **885** | 0 | 1 | 0 |  |
| **20** | 0 | 1 | 0 | 0 | 8 | 26 | 0 | 0 | 13 | 1 | 1 | 0 | 2 | 1 | 0 | 0 | 0 | 0 | 4 | 839 | 3 | 1 |  |
| **21** | 0 | 0 | 14 | 1 | 6 | 6 | 4 | 0 | 12 | 8 | 1 | 16 | 37 | 0 | 2 | 1 | 0 | 0 | 0 | 3 | **788** | 1 |  |
| **22** | 0 | 1 | 0 | 0 | 0 | 0 | 0 | 0 | 1 | 0 | 0 | 1 | 0 | 4 | 1 | 9 | 0 | 0 | 8 | 1 | 1 | 873 |  |
|  |  |  |  |  |  |  |  |  |  |  |  |  |  |  |  |  |  |  |  |  |  |  |  |
| Missclassified: | | | |  |  |  |  |  |  |  |  |  |  |  |  |  |  |  |  |  |  |  |  |
|  | 28 | 16 | 72 | 39 | 68 | 77 | 53 | 32 | 123 | 96 | **8** | 71 | 77 | 76 | **141** | 62 | 30 | 24 | 62 | 24 | 77 | 49 |  |

|  | the highest values |
| --- | --- |
|  | the lowest values |
